# P-1133. Preferred (Spoken) Language was a Major Correlate of Disparate Antibiotic Prescribing in the Pediatric Outpatient Setting Throughout the COVID-19 Pandemic

**DOI:** 10.1093/ofid/ofae631.1320

**Published:** 2025-01-29

**Authors:** Melissa E Day, Qing Duan, Justin Markham, Joshua D Courter, Kimberly Risma, Andrew F Beck, David Haslam

**Affiliations:** Cincinnati Children's Hospital Medical Center, Cincinnati, Ohio; Cincinnati Children's Hospital Medical Center, Cincinnati, Ohio; Cincinnati Children's Hospital Medical Center, Cincinnati, Ohio; Cincinnati Children's Hospital Medical Center, Cincinnati, Ohio; Cincinnati Children's Hospital Medical Center, Cincinnati, Ohio; Cincinnati Children's Hospital Medical Center, Cincinnati, Ohio; Cincinnati Children's Hospital Medical Center, Cincinnati, Ohio

## Abstract

**Background:**

No studies have explored sociodemographic variation in antibiotic prescribing across phases of the COVID-19 pandemic. We explored relationships between race, language, socioeconomic measures, and outpatient antibiotic prescribing patterns at our institution before and during the pandemic.

**Figure:**
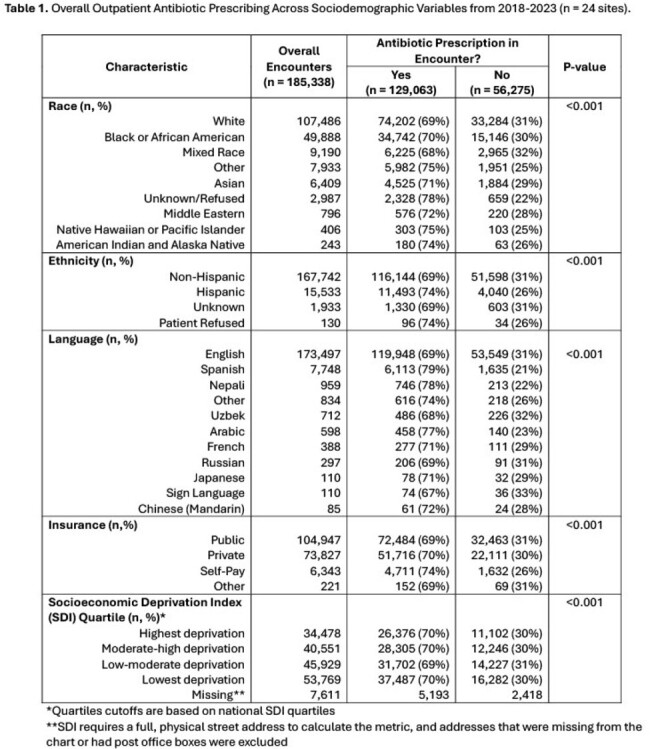

**Methods:**

Data were obtained across all Cincinnati Children’s outpatient primary care centers, urgent cares, and emergency departments (n = 24) from 2018-2023. Outpatients aged 0-18 years with an acute respiratory tract infection were included. Our primary outcomes were the proportion of encounters with antibiotics prescribed. Sociodemographic factors included race, ethnicity, primary language, insurance status, and a socioeconomic deprivation index (SDI) linked to a child’s geocoded home address (with higher scores showing more deprivation). We examined associations between such factors and antibiotic prescribing before (2018-19) and during (2020-21 and 2022-23) the COVID-19 pandemic. Chi-square analyses evaluated associations of proportions across groups.

**Figure:**
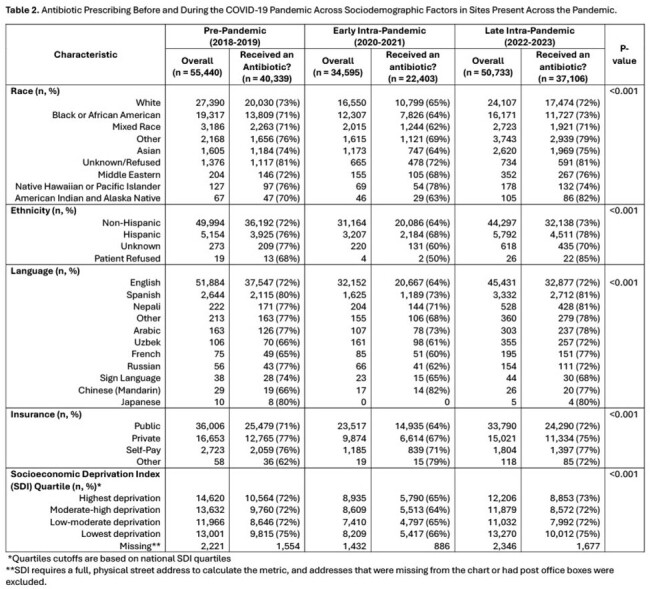

**Results:**

A total of 185,338 clinical encounters of 99,037 children with median age 3.6 years (IQR 1.3-7.5), 58% White, 9% Hispanic, and 93% English-speaking were captured from 2018-2023. **Table 1** shows associations of sociodemographic variables and antibiotic prescribing. While only small differences were noted in prescribing across race and insurance, patients preferring languages other than English had significantly more antibiotic use (Spanish (79%), Nepali (78%), Arabic (77%), Other (74%)) than those who preferred English (69%, p< 0.001). No clinically significant differences (≥5%) were found across SDI quartiles. Antibiotic use decreased early in the COVID-19 pandemic among all sociodemographic groups, rebounding to or above prior pre-pandemic levels and maintaining sociodemographic differences later in the pandemic (**Table 2**).

**Conclusion:**

At our institution, contrary to prior studies, children who preferred languages other than English received more antibiotics than English-speaking peers, associations which persisted across the COVID-19 pandemic. Next steps include investigating language-based differences in infectious diagnoses and antibiotic prescribing across individual outpatient sites.

**Disclosures:**

**All Authors**: No reported disclosures

